# Fatal attraction: intestinal amebiasis and COVID-19 as risk factors for colonic perforation

**DOI:** 10.1093/jscr/rjab301

**Published:** 2021-07-23

**Authors:** Jorge Alberto Dorantes, Jose Octavio López-Becerril, Maria G Zavala-Cerna

**Affiliations:** International Program of Medicine, Universidad Autónoma de Guadalajara, Zapopan, Jalisco, México; Departamento de Cirugía, Hospital General de Tijuana, Tijuana, BC, Mexico; Immunology Research Laboratory, Universidad Autonoma de Guadalajara, Zapopan, Jalisco, México

**Keywords:** intestinal amebiasis, hematochezia, colonic perforation, COVID-19, organotropism, thrombosis

## Abstract

The parasite *Entamoeba histolytica*, the causal agent of amebiasis, is considered a worldwide emergent disease and still represents an important cause of death in Mexico. Here, we describe a clinical case, involving an inflammatory response to both Coronavirus Infectious Disease 2019 (COVID-19) and intestinal amebiasis 54-year-old, COVID-positive Mexican gentleman was admitted to surgery following 6 days of hematochezia. An exploratory laparotomy and colonoscopy revealed multiple fibrous and amebic ulcerations (5–10 cm in diameter), with necrotic tissue predominantly localized in the sigmoid, descending and ascending colon. We discuss the pathophysiological interplay of both COVID-19 and intestinal amebiasis with the aim of highlighting a potentially novel aggravating mechanism in surgical patients suffering from colonic perforation in the setting of abdominal sepsis.

## INTRODUCTION

Amoebic dysentery is a common health condition throughout the developing world. In Mexico, still ranks among the 20 most common causes of death [1]. According to the WHO, amebiasis causes 40 000–100 000 deaths annually [2]. The major culprit is *Entamoeba histolytica,* an anaerobic protozoan spread through fecal–oral transmission. Pathogenicity includes adherence to colonic mucins and colonization of the large intestine. Several risk factors, including immune microbial interactions, genetic susceptibility and malnutrition, can predispose individuals to severe disease [3, 4]. Clinical features of amebiasis can range from mild diarrhea, blood and mucus in the stool to acute fulminant necrotizing colitis. Occasionally, parasites travel through the portal vein to the liver and induce the formation of abscess [5].

The novel Coronavirus Infectious Disease 2019 (COVID-19) caused by SARS-CoV-2 is known to preferentially infect the cells of the respiratory system; however, recent studies suggest the virus has an affinity for other organs, suggesting that ‘organotropism influences the course of COVID-19 disease and, possibly, aggravates pre-existing conditions’ [6]. The present case report aims to describe the clinicopathological changes seen in a patient with both intestinal amebiasis and COVID-19 and calls attention to the importance of recognizing high-risk patients who may benefit from a specialized and careful evaluation before surgery.

**Figure 1:**
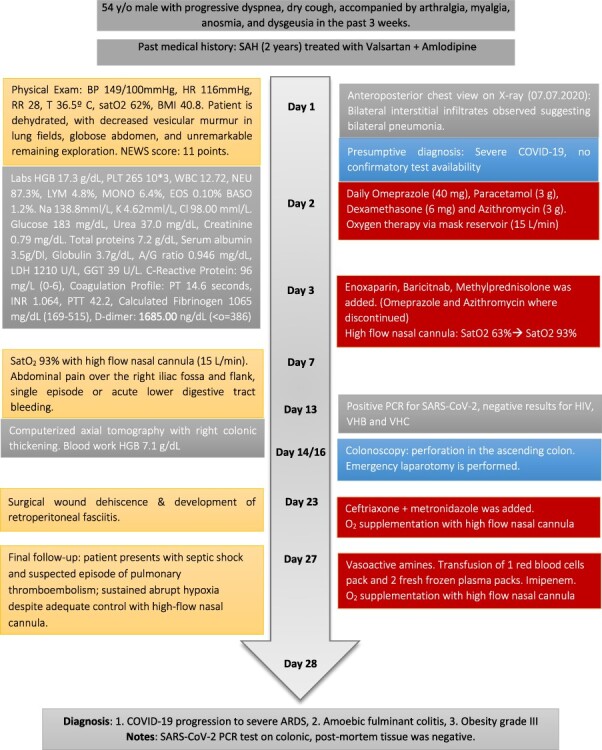
Patient’s timeline; SAH, systemic arterial hypertension.

## CASE REPORT

**Figure 2:**
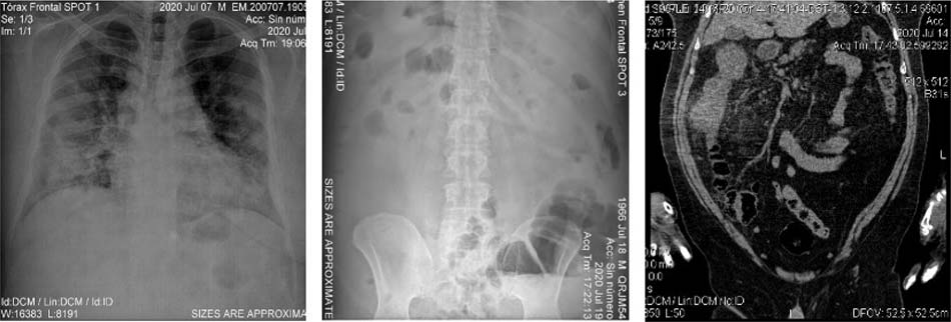
Imaging studies; (**A**) patient’s chest X-ray with bilobular diffuse pulmonary and alveolar infiltrate along with multiple opacities are present; (**B**) patient’s abdominal X-ray with dilated small intestine and colon with an occlusion located at the hepatic angle; edema and abdominal air fluid levels are noted; (**C**) abdominal CT scan showing engrossment of the ascending colonic wall at the hepatic angle with inflammation of pericolic adipose tissue; no free air or liquids are appreciated, suggesting a colonic occlusion.

**Figure 3:**
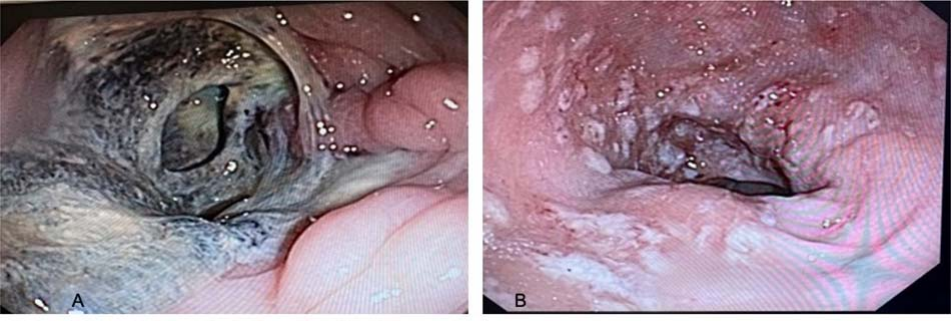
Colonoscopy views of the patient’s colon; (**A**) ascending colon with circumferential lesions and mucous membrane surrounded by necrotic and fibrotic tissue; (**B**) sigmoid colon and rectum with multiple ulcerated lesions observed, with depositions of fibrin from 5 to 10 mm in diameter, which represents the classic ‘flask-shaped lesions’ characteristic of amoebic colitis.

**Figure 4:**
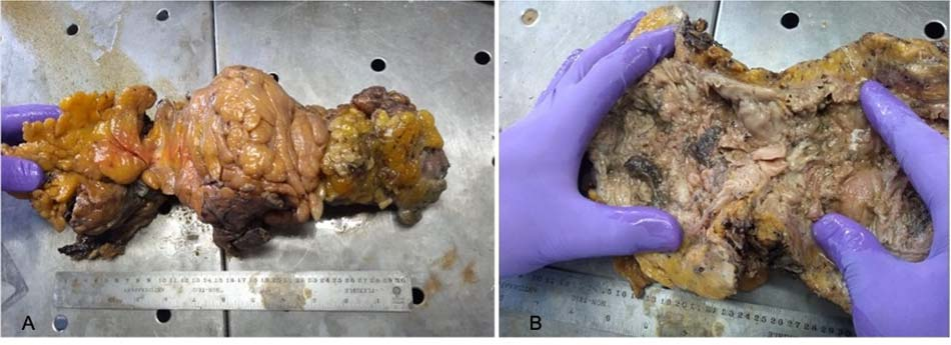
Macroscopic images of patient’s ascending colon; (**A**) ascending colon surrounded by epiploic appendages and inflamed adipose tissue; (**B**) ascending colon showing circumferential necrotic and fibrous tissue deposition.

**Figure 5:**
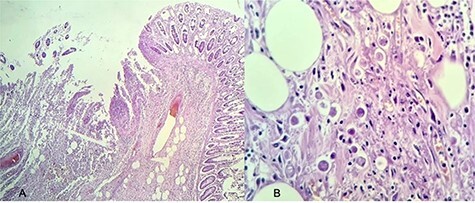
Histopathology of the patient’s resected colon; (**A**) intestinal crypts with multiple neutrophilic infiltrate (stained with hematoxylin and eosin, magnification 20×); (**B**) presence of multiple *E. histolytica* trophozoites (stained with hematoxylin and eosin, magnification 40×).

A 54-year-old male with a 2-year history of hypertension presented to the ER with dyspnea, anosmia and dysgeusia. Oxygen saturation (SatO2) was 62%, blood pressure (BP) was 149/100 mmHg and temperature was 35.6°. A detailed lab analysis is shown in Fig. 1. Due to COVID-19 suspicion, the patient was administered with paracetamol (3 g), dexamethasone (6 mg), plus oxygen therapy via mask reservoir (15 l/min). Later, he was admitted and started on high-flow oxygen therapy (Day 2), with the addition of daily enoxaparin (60 mg), baricitnab (4 mg) and methylprednisolone (80 mg). After 7 days, the patient developed severe abdominal pain localized to the iliac fossa and was referred for a surgical consultation (Day 9). A computed tomography (CT) abdomen showed hepatic steatosis without anatomical alterations in other organs. The visible portion of the thorax demonstrated a pattern of bilateral interstitial infiltrates, which is consistent with bilateral pneumonia (Fig. 2). An ultrasound (US) of the biliary ducts ruled out chronic cholecystitis or acute cholelithiasis. Three days later, the patient presented hematochezia and a reduction in hemoglobin (7.1 g/dl) (Day 12). A transfusion of packed red blood cells was started. A new abdominal US showed expansion of the ascending colon with mural engrossment without effusions. COVID-19-positive polymerase chain reaction (PCR) was reported on this same day (Day 13). A colonoscopy performed on Day 14 showed multiple ulcerations covered with fibrin (Figs 3 and 4). The patient was prepared for surgery and started on ceftriaxone (2 g) and metronidazole (1.5 g). An exploratory laparotomy performed on Day 16 revealed necrotic tissue and perforations of the ascending colon and retroperitoneal fasciitis. A right (stapled) hemicolectomy and ileostomy were completed. Vitals taken after surgery, the readings were as follows: SatO2: 80%, BP: 107/69 and temperature: 37.0°. Immediately after surgery, retroperitoneal purulent drainage was observed, which continued during his post-surgical stay. Blood work on Day 17 revealed fibrinogen and D-dimers, indicating a hypercoagulable state and signs of septic shock and neurological deterioration. After 7 days of the surgery (Day 23), the patient suffered wound dehiscence with erythematous markings and edema localized to his right flank. Blood work taken on that same day is shown in Fig. 1. A pack of red blood cells and two packs of fresh frozen plasma were transfused. Antibiotic treatment was changed to IV imipenem 2 g per day. Regrettably, the preceding interventions were not met with success, and after 28 days of hospitalization, the patient expired from sepsis-related complications. A portion of the colonic tract was preserved for histopathological analysis (Fig. 5).

## DISCUSSION

The combined presentation of hematochezia and elevated D-dimer levels in the setting of amoebic colitis and COVID-19 makes this an unusually challenging clinical encounter and surgical case. Typically, patients with extensive ulcerations disposed to heavy blood loss can have a favorable outcome in the operating room if infection is avoided. What makes this case uniquely intriguing is the concomitant interplay of immunological events characterized by both illnesses. It is highly probable that the pathological conditions of this gentleman’s pre-existing amoebic colitis were aggravated by SARS-CoV-2 infection.

The induction of tissue damage in *E. histolytica* initiates once trophozoites adhere to colonic epithelial cells, via the Gal-/GalNAc-specific lectin, whereby a lateral invasion gives rise to the classic flask-shaped ulcerations of amebiasis. After adhesion, receptors (TLR2/TLR4) expressed in the surface of both intestinal epithelial cells and resident macrophages recognize the carbohydrate domain of the lectin and induce the canonical pathway for inflammation [7], including interleukin (IL)-1B and COX-2, to synthesize PGE2 and IL-8 [8], involved in the chemoattraction of neutrophils and monocytes. Activation of these cells results in the production of ROS and tumor necrosis factor (TNF), which contributes to tissue damage and abscess formation [3, 9]. Approximately, 50 cysteine proteinases from *E. histolytica* have been implicated in triggering an inflammatory response in the gut and evasion of immunological mechanisms [10, 11], limiting the host immune response.

Emerging evidence of isolated SARS-CoV-2 from fecal samples suggests an enteric involvement resulting in GI symptoms prevalent in 17.6–61% of patients [12]. The expression of two mucosa-specific serine proteases (TMPRSS2 and TMPRSS4) promotes virus entry into host cells [13]. GI mucosal immunity to SARS-CoV-2 points toward the induction of a tolerogenic type of response [14], which is characterized by downregulation of inflammatory cytokine and chemokines in the intestines when compared to elevated cytokine activity in the lungs of patients with COVID-19 [15].

In cases of severe COVID-19, a significant elevation of IL-6 and D-dimers was associated with sepsis, coagulopathies and increased mortality [16].

In our case, it is complicated to determine to what extent SARS-CoV-2 infection was a trigger for associated coagulopathies, and this dilemma materialized in the contraindication of corticosteroids for intestinal amebiasis due to increased incidence of perforation in intestinal lesions [17]. Nevertheless, corticosteroids are among the limited treatments for SARS-CoV-2 to avoid progressive disease, and current guidelines for gastrointestinal procedures in COVID-19-positive patients do not address implications for surgical interventions that require time-sensitive procedures [18], like the one presented in this report.

## CONCLUSION

This case report represents a considerable gap in the knowledge of gut mucosal immunity, eliciting distinctive inflammatory profiles in an organ dependent manner and further complicating a pre-existing condition, namely *E. histolytica* infection. It is our purpose that this report can be used as an example to better inform surgical teams who are facing the unprecedented demands of this global pandemic since the underlying conditions diagnosed with SARS-CoV-2 may represent a veritable challenge for the patient’s treatment plans. We are optimistic that the scientific and medical communities can proactively advance the development of future solutions and perioperative protocols targeted to aid the most vulnerable patients in developing countries.
